# *Salmonella enterica *bacteraemia: a multi-national population-based cohort study

**DOI:** 10.1186/1471-2334-10-95

**Published:** 2010-04-14

**Authors:** Kevin B Laupland, Henrik C Schønheyder, Karina J Kennedy, Outi Lyytikäinen, Louis Valiquette, John Galbraith, Peter Collignon, the International Bacteremia Surveillance Collaborative

**Affiliations:** 1 Departments of Medicine and Pathology and Laboratory Medicine, University of Calgary and Calgary Laboratory Services, Calgary, Alberta, Canada; 2 Department of Clinical Microbiology, Aalborg Hospital, Aarhus University Hospital, Aalborg, Denmark; 3 Infectious Diseases Unit and Microbiology Department, The Canberra Hospital and School of Clinical Medicine, Australian National University, Woden, Australian Capital Territory, Australia; 4 Department of Infectious Disease Epidemiology, Hospital Infection Program, National Public Health Institute, Helsinki, Finland; 5 Department of Microbiology-Infectious Diseases, Université de Sherbrooke, Sherbrooke, Québec, Canada; 6 Microbiology Laboratory, Vancouver Island Health Authority, Royal Jubilee Hospital, Victoria, British Columbia, Canada

## Abstract

**Background:**

*Salmonella enterica *is an important emerging cause of invasive infections worldwide. However, population-based data are limited. The objective of this study was to define the occurrence of *S. enterica *bacteremia in a large international population and to evaluate temporal and regional differences.

**Methods:**

We conducted population-based laboratory surveillance for all salmonella bacteremias in six regions (annual population at risk 7.7 million residents) in Finland, Australia, Denmark, and Canada during 2000-2007.

**Results:**

A total of 622 cases were identified for an annual incidence of 1.02 per 100,000 population. The incidence of typhoidal (serotypes Typhi and Paratyphi) and non-typhoidal (other serotypes) disease was 0.21 and 0.81 per 100,000/year. There was major regional and moderate seasonal and year to year variability with an increased incidence observed in the latter years of the study related principally to increasing rates of non-typhoidal salmonella bacteremias. Advancing age and male gender were significant risk factors for acquiring non-typhoidal salmonella bacteremia. In contrast, typhoidal salmonella bacteremia showed a decreasing incidence with advancing age and no gender-related excess risk.

**Conclusions:**

*Salmonella enterica *is an important emerging pathogen and regional determinants of risk merits further investigation.

## Background

*Salmonella enterica *is a major cause of invasive infections worldwide [1-7]. Although a wide range of serotypes may cause human disease, they may be broadly grouped into the typhoidal species that are specific human pathogens and includes serotypes Typhi and Paratyphi, and other serotypes that are primarily spread to humans from animal sources (non-typhoidal). In high income countries, a major risk factor for acquiring typhoidal salmonella bacteremia is travel to an endemic region [8,9]. Foreign travel is also a risk factor for acquiring non-typhoidal salmonella infections, and several reports have highlighted the spread of resistant species globally [10,11]. However, non-typhoidal salmonella may frequently cause human disease in high income countries and in these cases risk factors include exposure to contaminated food, extremes of age, and the presence of a number of co-morbid illnesses [12-16].

In order to best establish the distribution and determinants of an infectious disease, population-based studies are optimal. This is because in these designs, selection bias is minimized by inclusion of all cases of disease fulfilling a case definition occurring among residents of a defined population. In addition, because the population at risk can be defined, incidence rates can be calculated that facilitate comparison among different populations and time periods. Only a few, relatively small studies have investigated the epidemiology of *S. enterica *bacteremia at the population level in single regions [17-19]. The objective of this study was to define the occurrence of *S. enterica *bacteremia in a large international population and to evaluate temporal and regional differences.

## Methods

### Study protocol and definitions

This study utilized a multi-centre population-based cohort design. All incident episodes of *S. enterica *bacteremia as defined by growth from one or more blood cultures from a resident of the surveillance population during January 1, 2000 to December 31, 2007 were identified. Year of culture, patient's age and gender were available for all cases and further microbiology and clinical information such as admission status were collected from all sites except Finland.

Bacteremia was defined as either hospital-onset (first occurred > 2 days after hospital admission) or community onset (first identified in community or within 2 days of hospital admission). Non-typhoidal salmonella infections were those exclusive of serotypes Typhi and Paratyphi. Only the first episode of salmonella bacteremia serotype per patient per year was included. Each center received approval from their local ethical review committee.

### Surveillance populations

Surveillance was conducted in Denmark, Finland, Australia, and three regions in Canada under the auspices of the International Bacteremia Surveillance Collaborative [20]. Microbiology laboratory testing was performed by each region according to their local procedures. Data were abstracted from each region using a standardized template. The Danish surveillance region was the North Denmark Region using the previous boundaries of the North Jutland County (population 495,000). Data were obtained using the North Jutland Bacteremia Research Database administered by the Department of Clinical Microbiology at Aalborg Hospital [21]. Surveillance data from Finland (population 5.3 million) was obtained using the National Infectious Disease Register (NIDR) to which all Finnish clinical microbiology laboratories report all bacterial isolations from blood. The Canberra Region (population 370,000) includes the city of Canberra within the Australian Capital Territory and the satellite city of Queanbeyan and several small surrounding rural towns within the state of New South Wales. Surveillance was conducted in three of the four microbiology laboratories that are estimated to capture more than 95% of all positive blood cultures in the region [22]. The three Canadian centers included Sherbrooke, Quebec, Victoria, British Columbia, and Calgary, Alberta. Sherbrooke has a total population of 152,000 residents and is served by a single microbiology laboratory located in the Centre Hospitalier Universitaire de Sherbrooke [23]. Data from the Victoria area included the south local health area of the Vancouver Island Health Authority (population 364,000), and data were obtained from the regional microbiology laboratory [20]. Laboratory based surveillance in the Calgary Health Region (population 1.2 million) was conducted at Calgary Laboratory Services that performs virtually all of the blood culture testing among residents of the region [24].

### Data management and statistical analysis

Data from each of the surveillance centres was compiled and analysed using Stata version 10.0 (Stata Corp, College Station, TX). Prior to description of analysis, all continuous variables were plotted using histograms to assess their underlying distribution. Non-normally distributed variables were reported as medians with inter-quartile ranges (IQR). The incidence of salmonella bacteremia was calculated by dividing the number of incident cases by the surveillance population as determined by census data from each of the surveillance areas. Incidence rates overall and for non-typhoidal and typhoidal salmonella bacteremia were directly standardized by age deciles and gender to the 2007 27-country European Union (EU27) population and reported as rates per 100,000 inhabitants [20]. Because of the small numbers in many cells, crude incidence rates were reported in sub-group analyses. Risks in population groups were expressed as incidence rate ratios (RR) and reported with 95% confidence intervals.

## Results

During the 8-year study, a total of 622 incident *S. enterica *bacteremias occurred during 61,748,478 person-years of surveillance; 370 (59%), 107 (17%), 78 (13%), 27 (4%), 21 (3%) and 19 (3%) cases were from Finland, Calgary, North Denmark, Sherbrooke, Victoria, and Canberra, respectively. Non-typhoidal isolates accounted for 490 (79%) cases, and of the 132 typhoidal salmonella cases, 85 (64%) were serotype Typhi and 47 (36%) were serotype Paratyphi.

### Incidence

**Figure 1:**
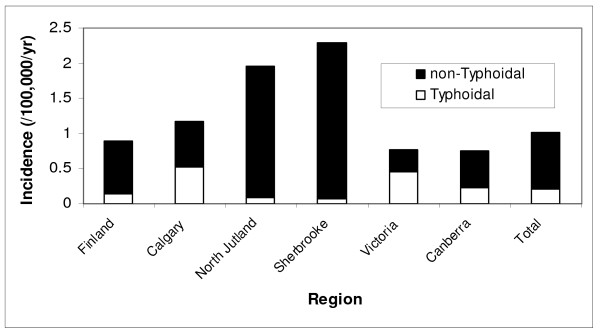
**Age and gender standardized incidence of salmonella bacteremia by region and group, 2000-2007**.

**Figure 2:**
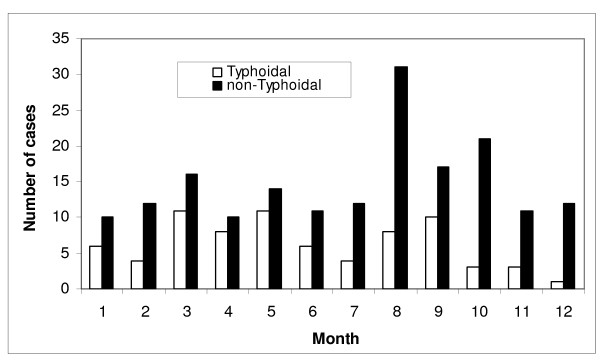
**Annual incidence of salmonella bacteremia, 2000-2007**.

**Figure 3:**
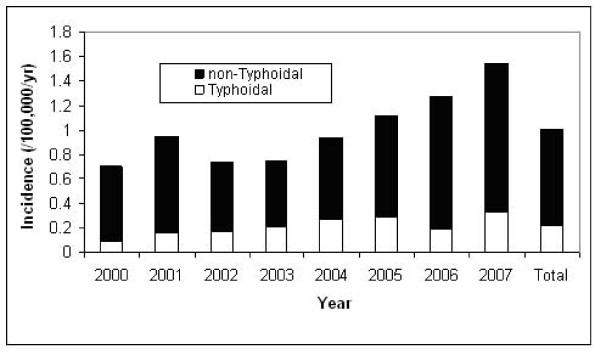
**Monthly occurrence of salmonella bacteremia (data from Finland not included)**. Month shown refers to month of the year (ie January = month 1) for northern hemisphere sites and adjusted by 6 months for opposite season in Australia (ie January = month 7).

The overall age and gender standardized annual incidence rate was 1.02 per 100,000 population, and was 0.21 and 0.81 per 100,000 for typhoidal and non-typhoidal bacteremia, respectively. The adjusted incidence rates were markedly different among the regions and by salmonella group as shown in Figure 1. There was moderate year to year variability with an overall increase in crude incidence observed in the latter years of the study related principally to increasing rates of non-typhoidal salmonella bacteremia as shown in Figure 2. The occurrence of salmonella bacteremia varied during the months of the year with the highest frequency in the late summer and early fall months as shown in Figure 3.

### Demographic factors

**Figure 4:**
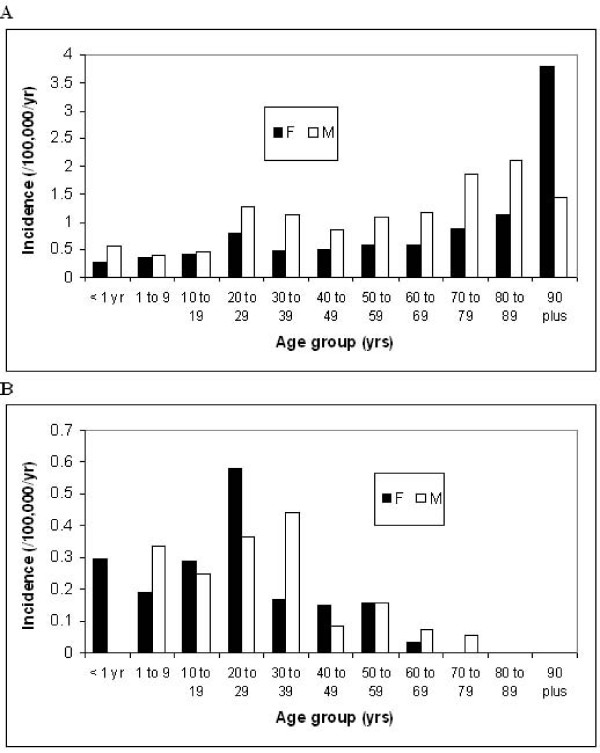
**Age and gender specific incidence of a) non-typhoidal and b) typhoidal salmonella bacteremia, 2000-2007**.

The overall risk of acquiring salmonella bacteremia varied significantly by age, gender, and salmonella group as shown in Figures 4a and 4b. Among non-typhoidal salmonella infections (Figure 4a), the risk increased with advancing age and males were at significantly higher risk (RR 1.62; 95% CI, 1.35-1.96; p < 0.0001). The excess risk for non-typhoidal infections in males was most notable among adults aged 70 to 89 (Figure 4a), with the markedly increased incidence of non-typhoidal salmonella bacteremia observed among aged 90 and older attributable to an excess number of cases in one centre (Denmark). In contrast, typhoidal salmonella bacteremias (Figure 4b) showed a decreasing incidence with advancing age and males were not at increased risk (RR 1.14; 95% CI, 0.80-1.62; p = 0.47).

### Clinical and microbiologic information

Further clinical and microbiology information was available for 252 cases. Among these, 234 (93%) were classified as community-onset and 204 (81%) were admitted to hospital for a median length of stay of 7 days (IQR, 4-11 days). Of the 18 hospital-onset cases, all were non-typhoidal serotypes and 10 (56%) were isolated during the first 3-5 days of admission. Of the remaining eight, the onset was 6, 8, 9 (2 cases), 15, 21 (2 cases) and 25 days after admission.

Among the 252 cases with added details, 55 (22%) were serotype Typhi, 20 (8%) were serotype Paratyphi, and 177 (70%) were non-typhoidal serotypes that included 36 *S*. Enteritidis, 22 *S*. Heidelberg, 20 *S*. Typhimurium, 8 *S*. Newport, 7 each of *S*. Dublin *and S*. Virchow, 6 *S*. Hadar, 4 each of *S*. Oranienburg and *S*. Saint Paul, 3 each of *S*. Java, *S*. Panama, *S*. Poona, *S*. Choleraesuis, and *S*. San Diego, 2 *S*. Arizonae, and one each of *S*. Muenster, *S*. Infantis, *S*. Matadi, *S*. Schwarzengrund, *S*. Bournemouth, *S*. Napoli, *S*. Bovismorbificans, *S*. Colindale, *S*. Haifa, *S*. Corvallis and *S*. Agona. Other isolates not identified to the specific serotype level included salmonella group C1/2 in 8, group D in 4, group B in 18, and were not reported in 5 cases.

Susceptibility testing results were available for 246 isolates. Reduced susceptibility to ampicillin was found in 52 (21%), and to ciprofloxacin in 7 (3%), and ceftriaxone in 7 (3%) cases. All isolates with reduced susceptibility to ceftriaxone were from Sherbrooke and 6/7 ciprofloxacin resistant isolates were from North Denmark.

## Discussion

This study reports the first novel results of the evolving International Bacteremia Surveillance Collaborative and provides new population-based information on the epidemiology of salmonella bacteremia. Our collaborative was initially formed in 2008 to address the lack of coordinated population based study of invasive bacterial diseases internationally [20]. Our present results highlight the value of such an initiative. The more than 60 million combined patient years of observation enabled us to study a large number of cases in low incidence areas. In addition, we were able to directly calculate the incidence of disease because in our surveillance areas all cases were included (sampling was not performed) and we had well-defined populations at risk for denominator data. This contrasts with non-population based studies such as hospital-based case series where sampling bias is a potentially major concern and the population at risk is rarely definable for community-onset disease [25]. Furthermore, by age and gender standardization to the EU27 population, we were able to meaningfully compare rates of disease in our surveillance regions. We observed that salmonella bacteremia is a relatively uncommon cause of bacteremia in our populations, but that considerable demographic and regional differences in occurrence exist.

The observations of significant differences among regions and by species group are important and novel. While we believe these represent true differences in incidence, a number of other factors could potentially at least in part explain these observations. Since the risk for salmonella bacteremia is influenced by age and gender, where populations differ in demographic structure then crude incidence rates may be expected to differ. However, we controlled for this possibility by age and gender standardizing our overall incidence rates. A second potential issue is that there may have been different rates of case ascertainment (identifying all culture-positive cases among residents) among our surveillance regions. While in some cases ascertainment was expected to approach or equal 100% such as with the national mandatory reporting system in Finland, this was not strictly the case in every other region [20]. However, all the other surveillance areas in this study are highly captive and estimated to be 95% or higher, such that the 2-3 fold differences in rates could not be explained by these small potential differential rates of ascertainment. A third possibility is that there may have been differences in the culturing practices among regions. This arises if the decision to sample blood for culture is significantly different among regions, and it may be expected that areas that sample more frequently may observe higher numbers of culture-positive cases. It is important to note that in all of our study regions the cost of blood culturing is fully publicly funded and available to all residents irrespective of financial means. While we cannot directly assess the issue of differential blood culturing pratices among our regions as we do not have data on negative cultures, it is notable that in previous individual publications on other common species causing bloodstream infections such as *Escherichia coli *and *Staphylococcus aureus*, similar crude incidence rates were reported [22-24,26,27]. Although we may speculate as to why the incidence of salmonella bacteremia differs so greatly among our regions, further studies are needed to explore factors such as travel, food preparation practices, agricultural, and other factors [8,28].

Our observation of increased risk for salmonella bacteremia during the summer has been previously well documented [17,29,30], and has been proposed to be due to increased travel abroad and lax food preparation related to outdoor cooking in that season. Several previous studies have documented increased colonization rates with salmonella species among food animals during the summer months [31-33]. Our observation of an increased risk for non-typhoidal salmonella bacteremia with advancing age is likely at least in part due to a higher prevalence of co-morbid illnesses in the older age group that predispose to bacteremia risk [25,34]. The excess population risk in males for non-typhoidal salmonella bacteremia is a novel finding. We speculate that this may be due to agricultural or diet exposure in males. Typhoidal salmonella bacteremia occurred in younger individuals and no gender-associated risk was evident. We suspect that this may reflect a greater rate of foreign travel to exotic typhoidal salmonella endemic regions in the younger age groups or possibly may reflect younger immigrant populations. However, while we speculate reasons as to why age and gender may modify risk, we do not have empiric data to support these suspicions. The determinants of increased risk for salmonella bacteremia in our surveillance regions merits further prospective investigation.

While this study benefits from the large, multi-national, population-based design, there are some limitations that merit discussion. The surveillance regions involved in this study are high income Western countries with largely publicly funded health systems. These results should therefore not be generalized to other dissimilar jurisdictions especially low income countries and tropical areas. In low income countries salmonella typically is among the most common bacterial pathogens and may be responsible for as many as one-half of all bacteremias [6,35,36] whereas they typically represent ≤1% in high income countries [18,37]. Incidence rates for typhoid in the developing world, especially the Indian subcontinent, may be several orders of magnitude higher than that reported here [7,36]. Second, the information that we collected was retrospective. While we used common criteria for inclusion procedures and data were abstracted using a standardized template, the routine laboratory testing procedures and methods of clinical data collection varied somewhat site-by-site. For example, only basic information was available from Finnish cases, and microbiology and clinical details from the other centers were determined using the local data sets and procedures. These included prospective review in Denmark and electronic records review and/or database extraction in other sites. In addition, ideally isolates would have been serotyped and tested for antimicrobial susceptibility in a single centralized laboratory. Finally, it would have been useful to have added information on other potential risk factors such as food and animal exposures, international travel, and co-morbid illness.

## Conclusions

In summary, in this multi-national collaborative study we report the epidemiology of salmonella bacteremia and find regional and temporal differences in occurrence. Further studies designed to define the determinants of risk for acquiring salmonella bacteremia are warranted. This report highlights the benefit of international co-operation in bacteremia surveillance and sets the stage for future collaborative studies.

## Competing interests

The authors declare that they have no competing interests.

## Authors' contributions

KBL contributed to data collection and conducted the primary analysis and drafting of the manuscript. HCS, KJK, OL, LV, JG, and PC contributed to data collection and manuscript preparation. All authors read and approved the manuscript.

## Pre-publication history

The pre-publication history for this paper can be accessed here:

http://www.biomedcentral.com/1471-2334/10/95/prepub
